# Effects of alpha fetoprotein on escape of Bel 7402 cells from attack of lymphocytes

**DOI:** 10.1186/1471-2407-5-96

**Published:** 2005-08-05

**Authors:** Mengsen Li, Xinhua Liu, Sheng Zhou, Pingfeng Li, Gang Li

**Affiliations:** 1 Department of Biochemistry and Molecular Biology, Peking University Health Science Center, Beijing 100083, China; 2 Department of Biochemistry, Hainan Medical College, Haikou 570102, China

## Abstract

**Background:**

Involvement of AFP against apoptosis of tumor cell has been implicated in its evasion of immune surveillance. However, the molecular events of immune escape mechanisms are still unknown. The major observations reported here relate to a possible mechanism by which heptoloma Bel 7402 cells escape immune surveillance *in vitro*.

**Methods:**

Western blotting and a well-characterized cofocal scanning image were performed to analyze the expression of Fas/FasL and caspase-3 in co-cultured Bel 7402 and Jurkat cells.

**Results:**

After co-culture with Jurkat cells, up-regulated Fas and reduced FasL expression could be observed. Treatment with AFP could remarkably inhibit the elevated Fas and, whereas, induce the FasL expression in co-cultured Bel 7402 cells. Cells co-culture could induce the expression of caspase-3 in both cells line. The elevated caspase-3 in Bel 7402 cells was abolished following the treatment of AFP. The expression of caspase-3 was elevated in co-cultured Jurkat cells treated with AFP. No detectable change on the expression of survivin was examined in both cells line. Monoclonal antibody against AFP treatment alone did not obviously influence the growth of cells, as well as the expression of Fas/FasL and caspase-3. However, the effect of AFP could be blocked by antibody.

**Conclusions:**

our results provide evidence that AFP could promote the escape of liver cancer cells from immune surveillance through blocking the caspase signal pathway of tumor cells and triggering the Fas/FasL interaction between tumor cells and lymphocytes.

## Background

Alpha fetoprotein (AFP) is one of several oncofetal proteins synthesized in large amounts by the fetus and drops in serum markedly shortly after birth. AFP as a tumor-associated fetal protein has demonstrated clinical utility as a tumor marker. Besides its role as a carrier or transporter for various serum ligands including fatty acids, retinoids, steroids, drugs, dyes and heavy metals, AFP has been reported to display growth regulatory properties. Previous studies have verified that AFP appears to functions as a growth regulator rather than only serum carrier. Multitude of cell types involving the growth and differentiation effects of AFP include placental [1], lymphoid [2], ovarian [3], uterine [4], gastric cancer [5], epidermal [6], breast cancer [7] and fetal fibroblasts [8]. Recently, some studies on the mechanisms of AFP suggested that AFP induced apoptosis in tumor cells independently of Fas/Fas ligand or TNFR/TNF signaling pathway, and AFP-mediated cell death involved activation of the effector caspase-3-like proteases, but was independent of upstream activation of the initiator caspase-1, caspase-8, and caspase-9-like proteases [9]. The intracellular mechanism of AFP involving to cAMP-PKA signaling pathway after its binding to different affinity receptors has been also reported [10].

Although the biological roles of the oncoembryonal protein AFP, including immunoregulatory functions in a variety of immune responses including the humoral and cell-mediated types, have been reviewed in detail, the evidences for the role of AFP in hepatoma cells escaping from host immune surveillance are still unknown [11,12]. In a recent study, AFP was used as an effective tumor rejection antigen to observe its effect in T-cell immune responses, implicating a gene therapy-based strategy for hepatoma cells [13]. However, the over-expression of AFP in human hepatoma cells is concurrent with aberrant growth manifestation. We presume that the altered serum AFP level is the cause of such changes rather than a coincident phenomenon and should be responsible for the malignant progression of liver cancer. Thus revealing the intracellular mechanisms underlying the evasion of tumor from host immune surveillance will provide further insights into the understanding for the biological role of AFP, particularly in the case of hepatocellular carcinomas.

## Methods

### Determination of cells proliferation

Jurkat T cells and Bel 7402 cells, the human hepatoma cell line, were adjusted to 3.0 × 10^4 ^per ml separately and maintained in PRMI-1640 medium supplemented with 10% heat-inactivated fetal bovine serum. The cells were seeded into 24-well plates and incubated at 37°C in a humidified atmosphere of 5% CO_2_. The supernatant was replaced to RPMI-1640 medium free serum for 24 h, then the various concentrations (5, 10, 20, 40, 80 or 100 mg/L) of AFP (Biodesign International Co. USA), human serum albumin (HSA, from Sigma, USA) and anti-AFP antibody (Santa Cruz. USA) were administrated into Jurkat T cells and Bel 7402 cells for 60 h respectively. The viability of cells was determined by Trypan blue exclusion assay.

### Cell co-culture assay

To observe the effect of AFP on the escape of tumor cell from the attack of lymphocytes, 1.5 × 10^4 ^of Jurkat calls and equal Bel 7402 cells that grew under such conditions were mixed and co-cultured onto 24-well plate. Following the incubation in RPMI-1640 medium for 24 h, AFP (20 mg/L), HSA (20 gm/L), AFP (20 mg/L) plus anti-AFP antibody (40 mg/L) and anti-AFP antibody (40 mg/L) were added into culture for 60 h. The fraction containing Jurkat cells were removed from flask by resuspending the supernatant gently and transferring the supernatant to a centrifuge tube. Bel 7402 cells in the bottom were scraped and collected. The viability of each cell line was determined by Trypan blue exclusion.

### Determination of Fas and FasL expression

Bel 7402 cells and Jurkat T cells were co-cultured as described above in Petri dish. AFP (20 mg/L), HSA (20 mg/L), AFP (20 mg/L) plus anti-AFP antibody (40 mg/L) and anti-AFP antibody (40 mg/L) were added into culture. At 48 hours treatment, Bel 7402 cells were washed 3 times with RPMI-1640 free serum to remove Jurkat cells. Cells were incubated with 0.5 ml of rabbit anti-Fas or anti-FasL solution (Santa Cruz Co, USA. 1:250 in RPMI-1640 medium) for 1 h. After washing with medium 3 times at room temperature, secondary goat anti-rabbit IgG antibodies conjugated with FITC (Santa Cruz, USA) were applied, and incubated for 1 hour at 37°C. After washing with PBS, the cells were observed under Confocal Laser Scanning Microscope (Leica TCS-NT SP2, Germany).

### Western blot immunodetection

1.5 × 10^4 ^Jurkat T cells and 1.5 × 10^4 ^Bel 7402 cells per ml were co-cultured in 75-cm^2 ^flasks and maintained in RPMI-1640 medium free serum for 24 h. To replace the supernatant with medium supplemented with 10% FCS, the supernatant which containing the Jurkat cells were removed, centrifuged and resuspended with fresh medium. The suspension was replaced into flasks to remix with Bel 7402 cells. Afterward, the co-cultured cells were treated with AFP (20 mg/L), HSA (20 gm/L), AFP (20 mg/L) plus anti-AFP antibody (40 mg/L) and anti-AFP antibody (40 mg/L) for 36 h respectively. Proteins were quantified before being loaded onto the gel. 40 μg of extracted proteins from each group was loaded onto 10% SDS polyacrylamide gels. Proteins were blotted onto a nitrocellulose membrane (Amersham, UK). Membranes were incubated for 1 h with anti-caspase-3 or anti-survivin monoclonal antibodies (Santa Cruz Co. USA) and then washed and revealed using anti-rabbit IgG horseradish peroxidase conjugate. Immunoreaction protein was detected by the chemiluminescence luminol reagent (Santa Cruz, USA).

### Statistical analyses

All experiments were performed at least three times. Data were presented as mean ± SD. The significance of the difference between experimental and control groups was analyzed with Student's *t *test. A value of *p *< 0.05 was considered to be statistically significant.

## Results

### The effects of AFP on the proliferation of cells

**Figure 1:**
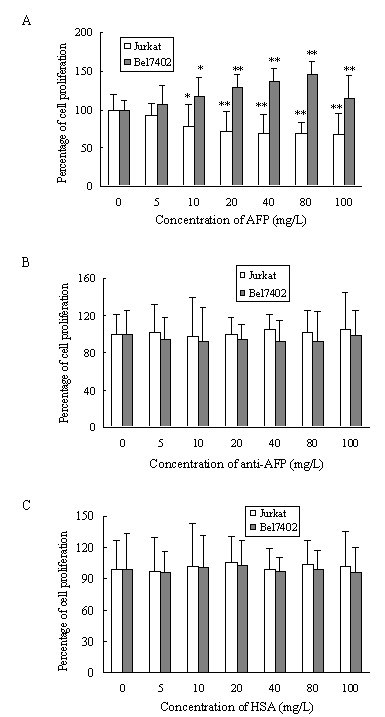
The effects of different concentrations of AFP on the growth of cells. The viability of Jurkat and Bel 7402 cells were observed after treated with different concentrations (5, 10, 20, 40, 80 or 100 mg/L) of either AFP (A), anti-AFP antibody (B) or HSA (C) for 60 h at 37°C in a humidified atmosphere of 5% CO_2_. *P < 0.05 and **P < 0.01 *vs *corresponding to normal control (0 mg/L) analyzed by *t*-test. Data were representative of an experiment that was repeated three times presented as mean ± SD of 12 samples.

AFP could enhance the proliferation of Bel 7402 cells in the dose-dependent manner in the concentration range of 10 to 80 mg/L (Fig 1A). The increment was up to 46.8% (80 mg/L) compared with control. However, the inhibited effect of AFP was observed in the growth of Jurkat cells. The maximum suppressive dose might be observed at the concentration of 80 mg/L (30.6% inhibition). As the controls, HSA and anti-AFP antibody did not display any detectable influences on the cell growth (Fig 1B and 1C).

### The effects of AFP on the growth of co-cultured cells

**Figure 2:**
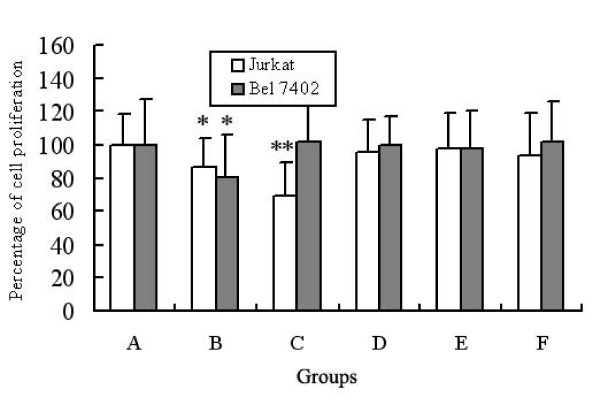
The effects of AFP on the co-cultured cells. Bel 7402 and Jurkat cells were co-cultured for 24 h following treated with AFP (20 mg/L) (C), AFP (20 mg/L) plus anti-AFP antibody (40 mg/L) (D), anti-AFP antibody (40 mg/L) (E) and HSA (20 gm/L) (F) respectively. Graph bar A represents the viability of separate-cultured cells. Graph bar B represents co-cultured and no-treatment group. After 60 h, two cell lines were separated and the viability of each cell was observed respectively. *P < 0.05 and **P < 0.01 *vs *corresponding to normal control (0 mg/L) analyzed by *t*-test. Data were representative of an experiment that was repeated three times presented as mean ± SD of 12 samples.

To observe the effects of AFP on the escape of Bel 7402 cells from the attack of lymphocytes, AFP at a concentration of 20 mg/L, which has been reported and proved to be an optimal dose according to the dose response curves in our recent experiments [unpublished data], was added into co-cultured cells. Jurkat and Bel 7402 cells were separated as described in *Materials and Methods *after the treatment of 60 h and the viability of each cell was determined. The results showed that the growths of both cells in untreated group were inhibited in the cells co-culture (Fig 2). However, the administration of AFP could obviously inhibit the proliferation of Jurkat cells, but not Bel 7402 cells after cells co-culture. Anti-AFP antibody could block the effect of AFP on cell proliferation. Antibody alone and HSA did not display any significant influences on the cell growth.

### Fas/FasL expression in Bel 7402

**Figure 3:**
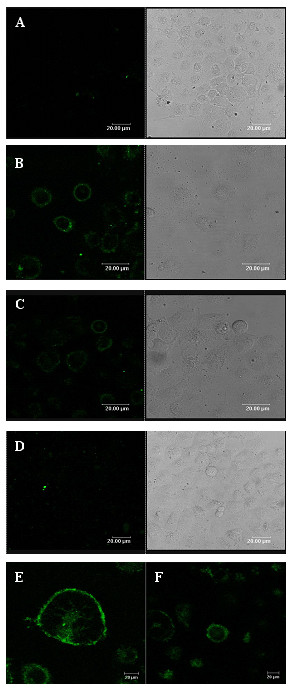
Confocal assay for the expression of Fas on the membrane of co-cultured Bel 7402 cells. Bel 7402 cells and Jurkat T cells were co-cultured with AFP (20 mg/L), anti-AFP antibody (40 mg/L), and AFP (20 mg/L) plus anti-AFP antibody (40 mg/L) respectively for 48 h. After removing Jurkat cells, Bel 7402 cells were incubated with rabbit anti-Fas antibody and secondary goat anti-rabbit IgG antibodies conjugated with FITC. The cells were observed using a confocal laser scanning microscope. Image A: separate-cultured Bel 7402 cell; B: separate-cultured Bel 7402 cells treated with AFP; C: co-cultured Bel 7402 cells treated with AFP; D: co-cultured Bel 7402 cells treated with anti-AFP antibody. Right images from A to D were taken under common microscope to observe the state of cells. E: co-cultured Bel 7402 cells; F: co-cultured Bel 7402 cells treated with AFP and anti-AFP antibody. All images were representative of an experiment that was repeated three times.

**Figure 4:**
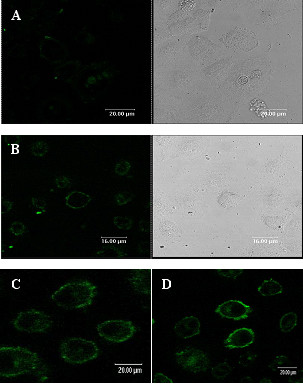
Confocal assay for the expression of FasL on the membrane of co-cultured Bel 7402 cells. Bel 7402 cells and Jurkat T cells were co-cultured with AFP (20 mg/L) for 48 h. After removing Jurkat cells, Bel 7402 cells were incubated with rabbit anti-FasL antibody and secondary goat anti-rabbit IgG antibodies conjugated with FITC. The cells were observed using a confocal laser scanning microscope. Image A: separate-cultured Bel 7402 cell; B: separate-cultured Bel 7402 cells treated with AFP. Right images of A and B were taken under common microscope to observe the state of cells. C: co-cultured Bel 7402 cells without AFP treatment; D: co-cultured Bel 7402 cells treated with AFP. All images were representative of an experiment that was repeated three times.

**Figure 5:**
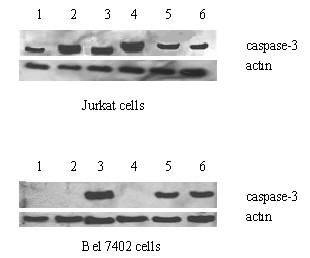
The effects of AFP on the expression of caspase-3 protein. Jurkat T cells and Bel 7402 cells were co-cultured for 24 h and treated with AFP (20 mg/L), HSA (20 gm/L), AFP (20 mg/L) plus anti-AFP antibody (40 mg/L) and anti-AFP antibody (40 mg/L) for 36 h respectively. The expression of caspase-3 was detected with Western blotting. Lane 1: control; Lane 2: cells separate-cultured cells treated with AFP; Lane 3: co-cultured cells; Lane 4: co-cultured cells treated with AFP; Lane 5: co-cultured cells treated with AFP and anti-AFP antibody; Lane 6: co-cultured cells treated with anti-AFP antibody. All images were representative of an experiment that was repeated three times.

AFP was added into either separate-cultured or co-cultured cells to observe its effects on the expression of Fas and FasL in Bel 7402 under confocal fluorescent microscope. As shown in Fig 5, untreated cells cultured separately did not exhibit green fluorescence on the surface of Bel 7402 (Fig 3A). After the administration of AFP for 48 h, only a diffuse image could be observed (Fig 3B). Bel 7402 cells co-cultured with Jurkat cells for 90 min exhibited an ever-increasing green fluorescence throughout the time course that reflected the presence of expressed Fas, which was faded after the addition of AFP (Fig 3E and 3C). Anti-AFP antibody alone did not change the fluorescent intensity of image and however, could partly reverse the effect of AFP on the expression of Fas (Fig 3D and 3F). In the observation of FasL, separate-cultured Bel 7402 cells did not display the expression of FasL (Fig 4A). The slight green outline of cells was observed after the addition of AFP (Fig 4B). The cell co-culture could induce the expression of FasL in Bel 7402 cells (Fig 4C). However, Bel 7402, in contrast with that observed in the assay of Fas, co-cultured with Jurkat cells in the presence of AFP could show an enhanced green fluorescent. A representative image is shown in Fig 4D.

### Immunodetection of apoptosis-related proteins

**Figure 6:**
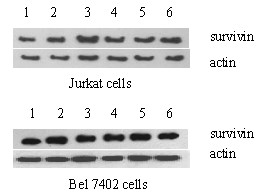
The effects of AFP on the expression of survivin protein. Jurkat T cells and Bel 7402 cells were co-cultured for 24 h and treated with AFP (20 mg/L), HSA (20 gm/L), AFP (20 mg/L) plus anti-AFP antibody (40 mg/L) and anti-AFP antibody (40 mg/L) for 36 h respectively. The expression of survivin was detected with Western blotting. Lane 1: control; Lane 2: cells separate-cultured cells treated with AFP; Lane 3: co-cultured cells; Lane 4: co-cultured cells treated with AFP; Lane 5: co-cultured cells treated with AFP and anti-AFP antibody; Lane 6: co-cultured cells treated with anti-AFP antibody. All images were representative of an experiment that was repeated three times.

When different cells were separately cultured, AFP could enhance the intracellular expression of caspase-3 in Jurkat cells and suppress that in Bel 7402 cells (Fig 5). In the co-culture experiment, the contents of caspase-3 in both cell lines were increased even though no AFP treatment. As shown by the intensities of the immuno-positive bands, the synthesis of intracellular caspase-3 in Jurkat cells was potently up-regulated after the administration of AFP. In contrast, no detectable change in the content of caspase-3 could be observed in the co-cultured Bel 7402 cells, indicating the block of AFP. The effect of AFP on the expression of caspase-3 in both cells could be abolished by anti-AFP antibody. As shown in Fig 6, AFP did not display significant influence on the level of survivin although a slight increment in co-cultured Jurkat cells.

## Discussion

During the last decade, it has been confirmed from a multitude of studies that AFP as a growth regulator modulates the ontogenic growth and tumor progression even though the overall findings remain controversial and their interpretations are still being debated. Previous studies also implicated that AFP expressed during pregnancy inhibits the maternal immune exclusion by suppressing immune responses of the humoral and cell-mediated types [14]. The higher level of AFP in the serum of liver cancer subject has been considered to be the reason of tumor development rather than merely the concomitant oncofetal protein. Hereby, the implication was emerged that AFP functions to constitute one of fundamental steps in the progression of hepatoma. It is in fact that one of mechanisms of progression and development of liver cancer is due to its escaping from immune surveillance. Experimental results obtained from the present study showed that AFP was capable of suppressing the growth of Jurkat cells with the concomitant proliferation of Bel 7402, indicating the diversity effects of AFP on the regulation of immune and tumor cells growth. This result was supported by a recent study, which indicated that AFP induced significant apoptosis of dendritic cells [15]. AFP-treated dendritic cells could produce low levels of IL-12 and TNF-α, a cytokine pattern that could hamper an efficient antitumor immune response. These results thereby offered a mechanism by which hepatocellular carcinoma escapes immunological control.

Co-culture experiment has been widely used for the determination of apoptosis, pathological response and tumor related protein synthesis [16-18]. The effects of Fas/FasL in the mechanism of tumor escaping from immune surveillance have been extensively documented [19-22]. It has been proposed that the expression of Fas/FasL in tumors may play a critical role in immune escape. In the present study, the expression of Fas/Fas L in target hepatoma Bel 7402 cells was examined after co-culture with the effector Jurkat cells *in vitro*. Our data showed that the expression of membrane Fas was enhanced in Bel 7402 cells co-cultured with Jurkat cells. The fact that the enhanced expression of Fas was abolished by AFP exposure indicated that the stimulation of Jurkat cells to Bel 7402 cells was able to be blocked by AFP. This is in accordance with our recent findings from immunodetection analysis (unpublished data). On the other hand, AFP enhanced the synthesis of FasL in Bel 7402 cells, which was consistent with previous findings [23-27]. AFP-induced over-expression of Fas on the surface of lymphocytes, together with simultaneous over-secreted FasL from tumor cells, could be one of reasons to accelerate the death of lymphocytes and facilitate the immune escape of liver cancer. This conclusion is supported by a recent study, which indicated that Fas-mediated apoptosis resulted by cancers expressing FasL and killing lymphocytes was irrespective of transforming growth factor-beta1 (TGF-beta1) expression [28].

Although the precise mechanism of AFP-mediated cell growth regulation and apoptosis induction remains obscure, there have been considerable investigations indicating that AFP can modulate apoptotic signals induced by various factors by either promoting or abrogating apoptosis. In the present study, the intracellular level of caspase-3 in Bel 7402 cells after co-culture with Jurkat cells was up-regulated, indicating the inducement of the apoptotic protein by lymphocytes. Furthermore, the pretreatment of AFP *in vitro *in co-cultured cells led to the full abolishment of caspase-3. Whereas, an increment at the level of caspase-3 protein was observed in co-cultured Jurkat cells after stimulated by AFP, which resulted in the depletion of lymphocytes in culture. The accelerated death of lymphocytes in current study might serve to certify the speculation that AFP-mediated apoptosis involved activation of the effector caspase-3-like proteases [9]. Therefore, it is reasonable to postulate that the escaping of tumor cells from immune surveillance is partly attributable to the apoptosis of lymphocytes induced by AFP. Taking into consideration the ability of AFP to alter the Fas/FasL expression in Bel 7402 and Jurkat cells, the signaling pathway involved might be initiated through the interaction of AFP with corresponding receptors and induced the activation of Fas/FasL and caspase-3 system. Our data elucidated that the death signal was triggered by activation of specific membrane AFP receptors involved in apoptosis signaling, leading to the expressional alteration of Fas/FasL and caspase-3. These findings are in accordance with the previous data showing that Fas/FasL was involved in AFP-induced immune escape [29-31].

## Conclusions

In summary, the present study has at least partly explained demonstrated the potential effect of AFP in malignant growth and transformation of liver tumor cell. Take together all findings, we propose that the effect of AFP in the escape of hepatoma Bel 7402 cells from immune surveillance is achieved though decreasing Fas in its membrane and secreting FasL which in turn to trigger apoptosis of lymphocytes via caspase-3 cascade. Thus, the involvement of Fas- and caspase-related signal pathway in the occurrence of liver tumor was thereby indicated in this study. The precise elucidation of the biological effect of AFP will help to better understand the regulatory mechanism of immune surveillance in liver cancer.

## Abbreviations

AFP: alpha fetoprotein; HSA: human serum albumin

## Competing interests

The author(s) declare that they have no competing interests.

## Authors' contributions

Mengsen Li and Xinhua Liu: carried out the study and contributed equally to this study.

Sheng Zhou: participated in the statistical analysis.

Pingfeng Li: conceived of the study, and participated in its design and coordination and helped to draft the manuscript.

Gang Li: conceived and design of the study, corresponding author.

## Pre-publication history

The pre-publication history for this paper can be accessed here:
